# A rapid inventory of amphibians, squamates, and bats of Mata de Plátano Field Station and Nature Reserve, Arecibo, Puerto Rico

**DOI:** 10.1002/ece3.11648

**Published:** 2024-07-23

**Authors:** Justin Matthew Bernstein, Camilo Andrés Calderón‐Acevedo, Pedro Ivo Mônico, Lázaro Willian Viñola‐Lopez, J. Angel Soto‐Centeno

**Affiliations:** ^1^ Center for Genomics University of Kansas Lawrence Kansas USA; ^2^ Smithsonian Tropical Research Institute Balboa Ancón Panama; ^3^ Department of Earth and Environmental Sciences Rutgers University‐Newark Newark New Jersey USA; ^4^ Department of Biology, Florida Museum of Natural History University of Florida Gainesville Florida USA; ^5^ Department of Mammalogy American Museum of Natural History New York New York USA

**Keywords:** bats, caves, frogs, karst, lizards, snakes, tropical moist forest

## Abstract

Puerto Rico harbors a diverse vertebrate fauna with high levels of endemism. However, while several books for vertebrate diversity and local checklists for birds have been published, checklists of amphibians, reptiles, and bats are lacking or nonexistent at both local and regional scales. In this study, we documented the amphibian, reptile, and bat faunas at Mata de Plátano Field Station and Nature Reserve, in Arecibo, Puerto Rico. We document four species of amphibians, seven lizards, three snakes, and nine bats. Despite prior works using samples from this nature reserve, this represents the first vertebrate checklist for the Mata de Plátano Field Station and Nature Reserve. We provide additional natural history observations based on our survey results and highlight the importance of including local and regional checklists of species for downstream research and conservation.

## Graphical abstract

We perform a rapid survey of amphibians, reptiles, and bats at the Mata de Plátano Field Station and Nature Reserve in Arecibo, Puerto Rico, a protected reserve adjacent to a larger forest. Our rapid survey resulted in finding 70%, 14%, and 22% of the amphibians, reptiles, and bat fauna, respectively, highlighting that smaller areas can act as refuges for a wide diversity of taxa and are important in regard to harboring ecologically important species and acting as functional ecosystems.

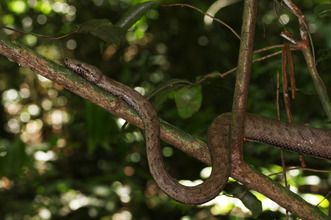

## 1 INTRODUCTION

**FIGURE 1:**
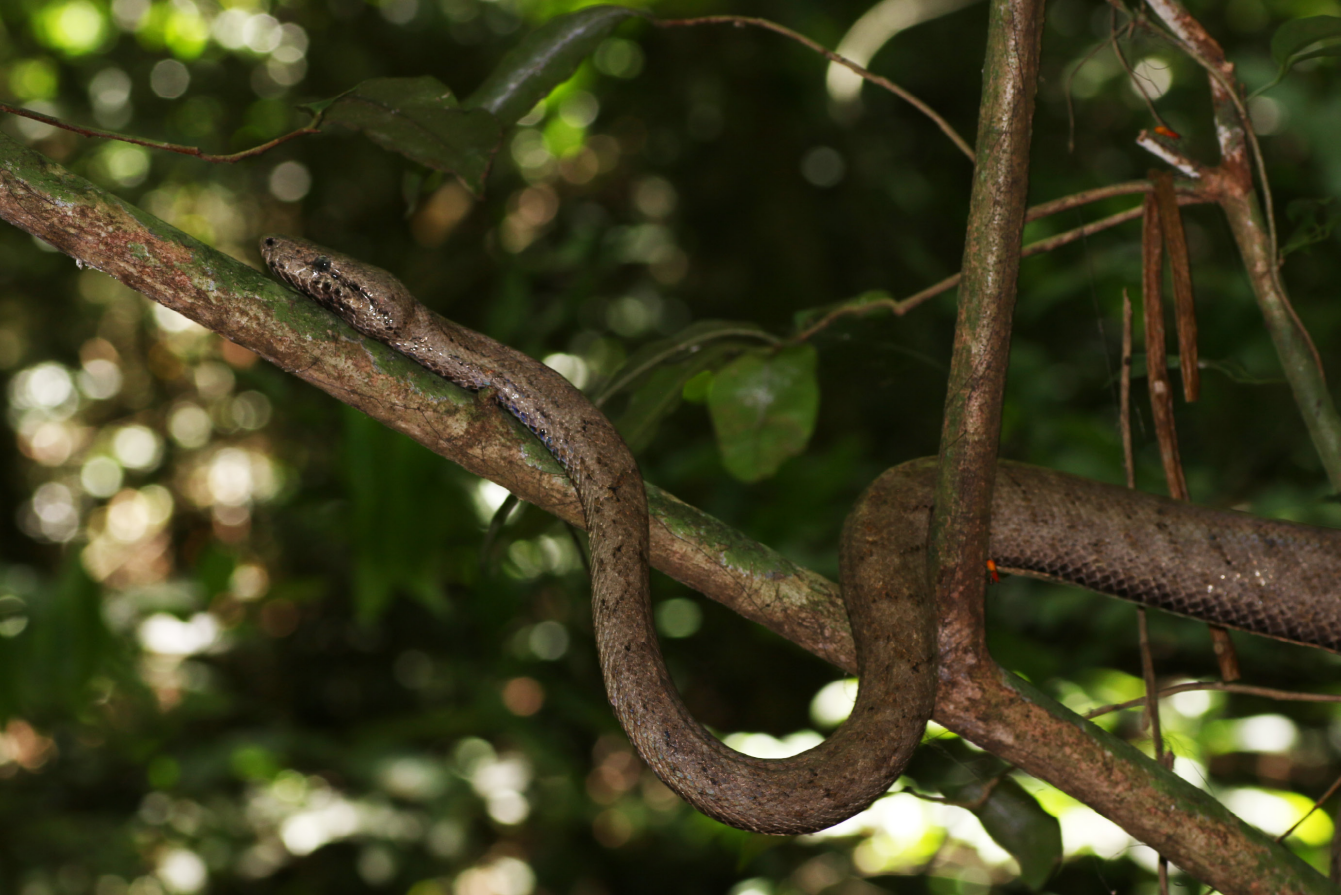
An individual of *Chilabothrus inornatus*, the Puerto Rican Boa, resting on a branch outside Cueva de los Culebrones.

Puerto Rico is an island of the Greater Antilles comprising ~9100 km^2^ and located at 18.2° N, 66.6° W between the Virgin Islands and Hispaniola. The island constitutes a small archipelago of over 125 islands and cays that are geologically complex. About 28% of the terrain of the main island of Puerto Rico is covered in limestone cliffs, valleys, and hills with a dichotomy between mesic and xeric forests in the northern and southern regions, respectively (Lugo et al., 2001; Monroe, 1976). The heterogeneity of Puerto Rico's landscape and habitats harbor diverse vertebrate fauna, especially in areas associated with forests and karst formations. Among the focal taxa in this study, the diversity of this island includes a total of 18 amphibians, 72 squamates (lizards and snakes) (Rivero, 1998; Uetz et al., 2023), and 13 bats (Gannon et al., 2005). For the herpetofauna, about 60 species (43%) are endemic to Puerto Rico; out of the bat species, 2 (15%) and 6 (46%) are locally endemic to Puerto Rico and regionally endemic to the West Indies, respectively. Nonetheless, distribution and taxonomic accounts of these taxa in locally protected habitats and preserves are generally lacking. Checklists at local and island‐wide scales for Puerto Rico are limited to algae (Ballantine & Aponte, 1997), insects (Ramírez et al., 2020), birds (Arendt et al., 2015), and arthropods (Ospina‐Sánchez et al., 2020; Pérez‐Reyes et al., 2013; Vélez Jr., 1967), but are lacking for bats, squamates, and amphibians. The need for more regional and up‐to‐date checklists in Puerto Rico is important for biogeographic, evolutionary (Wogan et al., 2020), and conservation studies (Ingram et al., 2022), as many taxa have restricted ranges and are isolated to regional caves and/or forests (Gannon et al., 2005; Rivero, 1998). Here, we provide the first faunal checklist for Mata de Plátano Field Station and Nature Reserve (collectively referred to as Mata de Plátano here), an area in the north‐central subtropical moist forests of Arecibo that contains multiple caves, including Cueva de los Culebrones, a cave system well‐known for the predator‐prey interactions between the endemic boa (Chilabothrus inornatus; Figure 1) and several bat species.

## 2 METHODS

### 2.1 Study area and sampling effort

**FIGURE 2:**
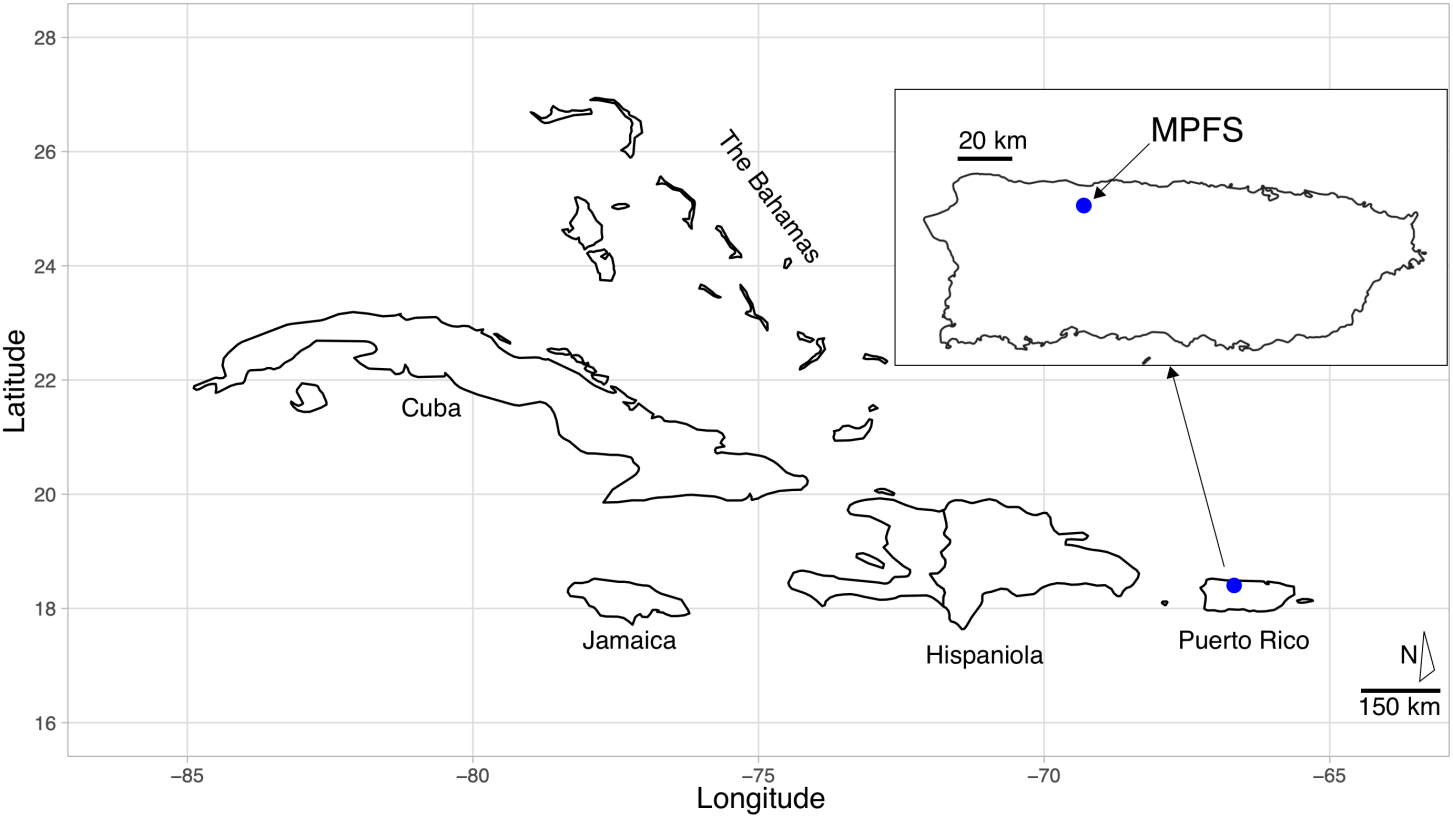
Map of the Greater Antilles and the Bahamas with Puerto Rico as the easternmost island. Inset represents the island of Puerto Rico, and the blue dot indicates the location of the Mata de Plátano Field Station (MPFS) in the municipality of Arecibo.

Mata de Plátano consists of a 53‐hectare, protected nature reserve, about 7 km southwest of the city of Arecibo. It is located in the northern karst belt region of Puerto Rico (18.414, −66.729) at 151 m above sea level (Figure 2). The reserve is part of the largest unfragmented and species‐rich forests of Puerto Rico. The sub‐tropical moist forested areas of the reserve consist of a few undisturbed areas among different successional stages of other forests. These areas have canopies up to ~20 m that receive precipitation that ranges from 100 to 220 cm/year, with dry months being from December–March, and wet months from April–November (Ewel & Whitmore, 1973). The study area in this checklist includes two cave systems: Cueva Larvas (18.414, −66.727) and Cueva de los Culebrones (18.414, −66.725). Cueva Larvas is a small cave with a wide entrance, while Cueva de los Culebrones is a 182‐meter (m) long hot cave with an entrance that is 5 × 3 × 8 (width × height × depth, m; Puente‐Rolón, 1999). Herpetofauna and bat occurrences were recorded during survey and mist net efforts in localities that were being explored (see Sections 2.2 and 2.3) for six survey days. Herpetofauna were captured using opportunistic sampling methods by looking in microhabitats (under logs, rocks, and in trees); all herpetofaunal occurrences in this study are observational and no individuals were collected.

### 2.2 Herpetofauna

Field surveying was conducted from 1 to 9 June 2022. Opportunistic sampling was performed to find lizards and snakes under logs, rocks, in leaf litter, on trees, and throughout cave systems. Most species were caught by hand, while other taxa (e.g., Puerto Rican Boas) were identified with photo vouchers and observed from a distance. Photographs were taken with a Nikon 70D DSLR camera with a Tamron AF 90 mm macro lens. Species identification was done using morphological criteria from Rivero (1998). No specimens were collected but were easily identifiable based on distinguishing morphology from syntopic taxa. We identified all species following Rivero (1998).

### 2.3 Bat fauna

Field surveys for bats were conducted during 1–9 June 2022. Bats were captured in mist nets (6–9 m long by 3 m high, Avinet 38 mm mesh) set along forest corridors near the field station. Mist nets were set after sunset on three nights, for 3 h each night (~19:00–22:00). Captured bats were identified to species, and standard measurements (i.e., forearm and weight) were taken. Photographs were taken with a Nikon 70D DSLR camera with a Tamron AF 90 mm macro lens and with a Cannon G12. No specimens were collected during surveys and all individuals were released at the site of capture. This study was conducted following the Departamento de Recursos Naturales de Puerto Rico (permit no. 2022‐IC‐034 issued to JASC) and under compliance with Rutgers IACUC (permit no. 201900087 issued to JASC). Coordinates for sites of capture were taken with a Garmin GPSMap 65 series to the nearest 0.001°. Identification of bats was based on Kurta and Rodríguez‐Durán (2023) (specific listings below, see Section 3).

### 2.4 Species accumulation curve

Although our sampling regime is over a short time period, we calculated a species accumulation curve (SAC) to determine if more sampling would be needed to obtain a higher species richness. This analysis was exclusively done for the herpetofauna, as sampling of bat fauna was done on only three nights, rather than daily for herpetofauna. We calculated the SAC using all known herpetofauna (introduced and native) from the sampling region in Arecibo, known from the CaribHerp database (http://www.caribherp.org/). We calculated our SAC using custom R scripts, modified from Sutton (2020), using the packages *pacman*.

## 3 RESULTS

During our field work, we found a total of 14 species of herpetofauna (four amphibians, seven lizards, and three snakes) and eight species of bats. We list our inventory of amphibians, reptiles, and bats below.

**Amphibians**

**Family Bufonidae**

*Rhinella marina* (Linnaeus, 1758)

Figure 3a

**FIGURE 3:**
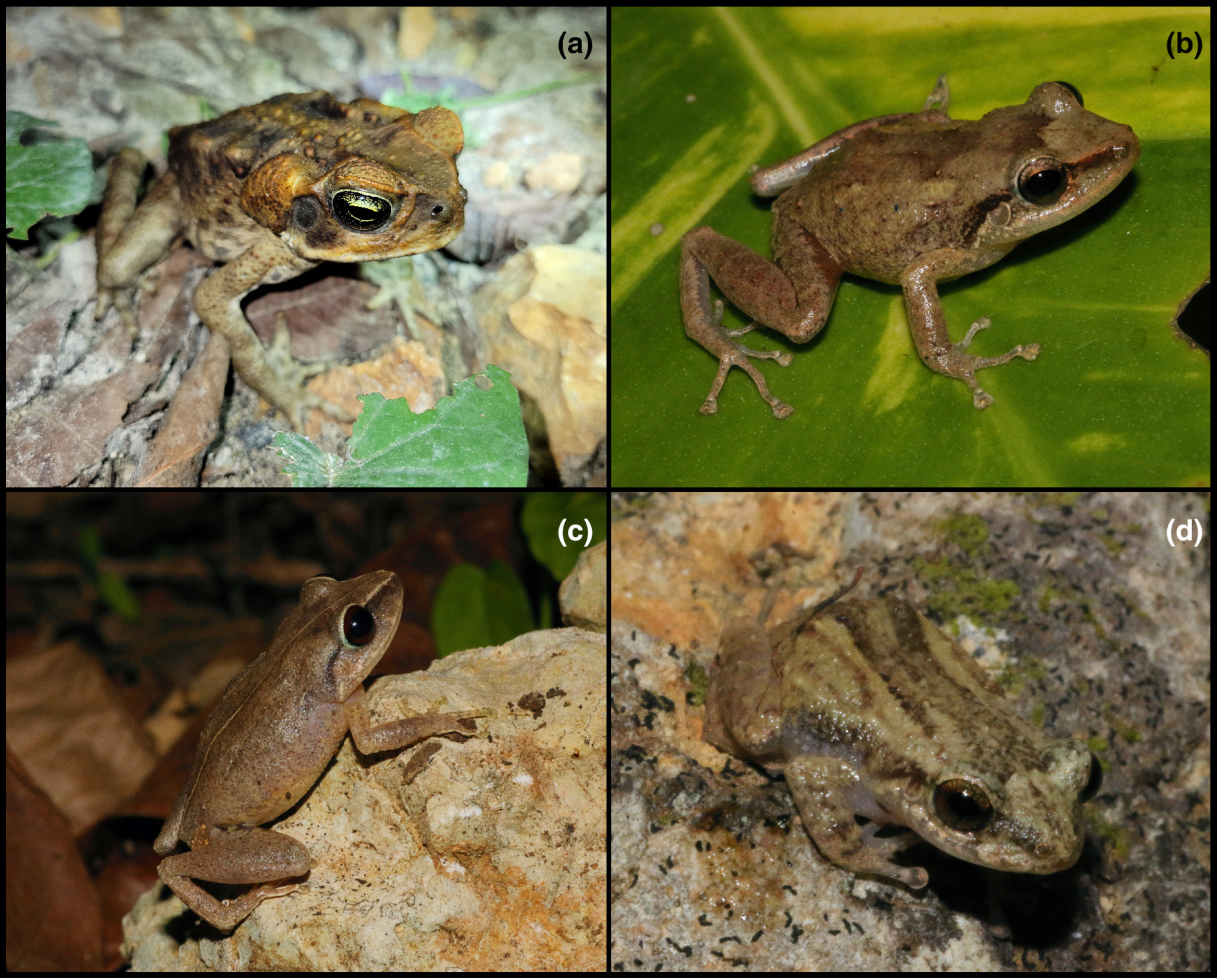
Frogs found at the Mata de Plátano Field Station and Nature Reserve. (a) *Rhinella marina*. (b) *Eleutherodactylus coqui*. (c) *Eleutherodactylus antillensis*. (d) *Eleutherodactylus cochranae*. All photographs taken by Justin M. Bernstein.

**Materials examined.** PUERTO RICO—**Arecibo** · Mata de Plátano Field Station and Nature Reserve; 18.414, −66.729; 151 m above sea level; Justin Matthew Bernstein, Camilo Andrés Calderón‐Acevedo, Pedro Ivo Mônico, Lázaro Willian Viñola‐Lopez, J. Angel Soto‐Centeno; Individual 1—found in leaf litter on south side of field station at 11:30 PM.

**Identification.** One adult individual, diagnosed by its large size, enlarged parotid glands, and drier, warty skin distinguishes this amphibian from all others on the island. It is easily distinguished from *Peltophryne lemur* by its shorter rostrum that is not upturned.

**Family Eleutherodactylidae**

*Eleutherodactylus coqui* Thomas, 1966

Figure 3b

**Materials examined.** PUERTO RICO—**Arecibo** · Mata de Plátano Field Station and Nature Reserve; 18.414, −66.729; 151 m above sea level; Justin Matthew Bernstein, Camilo Andrés Calderón‐Acevedo, Pedro Ivo Mônico, Lázaro Willian Viñola‐Lopez, J. Angel Soto‐Centeno; Individual 1—found in leaf litter on west side of field station at 6:00 PM; Individual 2—found on east wall of field station at 9:30 PM; Individual 3—found in leaf litter at far end of open field 73 m south of field station.

**Identification.** Three adults, identified by a combination of morphology and geographic distribution. Diagnosed by eye color (tan/brown) and color pattern (no dorsal stripe, saddle pattern on back and dorsolateral lines). *Eleutherodactylus monensis*, *E. cooki*, *E. cochranae*, *E. portoricensis*, *E. karlschmidti*, *E. gryllus*, *E. locustus*, *E. wightmanae*, *E. eneidae*, *E. richmondi*, *E. unicolori*, and *E. jasperi* are all outside the range of our study area. *Eleutherodactylus brittoni is* found in meadows and has a narrow black streak at the tip of the snout, stretching and continuing along the flanks (absent in our observed individuals). *Eleurtherodactylus portoricensis* has silver or chalky white eyes (tan/brown eyes in our individuals) and is restricted to mountains. *Eleutherodactylus hedricki* has a pronounced constriction behind the head and a shallow furrow on the middle of the back from the head to the sacrum (absent in our individuals). *Eleutherodactylus richmondi* has yellow or white lines on each side of the head, that range from the tip of the snout to near the vent (absent in our individuals). *Eleutherodactylus antillensis* has a dorsal stripe that stretches from the tip of the snout to the vent (absent in our individuals) and reddish eyes (tan/brown in our individuals).

*Eleutherodactylus antillensis* (Reinhardt and Lütken, 1863)

Figure 3c

**Materials examined.** PUERTO RICO—**Arecibo** · Mata de Plátano Field Station and Nature Reserve; 18.414, −66.729; 151 m above sea level; Justin Matthew Bernstein, Camilo Andrés Calderón‐Acevedo, Pedro Ivo Mônico, Lázaro Willian Viñola‐Lopez, J. Angel Soto‐Centeno; Individual 1—found in leaf litter near entrance of open field, 73 m south of field station; Individual 2—caught in mist net placed at path of open field 75 m south of field station 7:40 PM.

**Identification.** Two adults, identified by a combination of morphology and geographic distribution, were diagnosed by its thin, mid‐dorsal stripe on the head and body, and reddish eyes. Similar to the identification of *E. coqui*, the other species *E. monensis*, *E. cooki*, *E. cochranae*, *E. portoricensis*, *E. karlschmidti*, *E. gryllus*, *E. locustus*, *E. wightmanae*, *E. eneidae*, *E. richmondi*, *E. unicolori*, and *E. jasperi* are all outside the range of our study area. *Eleutherodactylus brittoni is* found in meadows and has a narrow black streak at the tip of the snout, stretching, and continuing along the flanks (absent in our observed individuals). *Eleurtherodactylus portoricensis* has silver or chalky white eyes (tan/brown eyes in our individuals) and restricted to mountains. *Eleutherodactylus hedricki* has a pronounced constriction behind the head and a shallow furrow on the middle of the back from the head to the sacrum (absent in our individuals). *Eleutherodactylus richmondi* has yellow or white lines on each side of the head, that range from the tip of the snout to near the vent (absent in our individuals). *Eleutherodactylus coqui* may or may not have the dorsal stripe that stretches from the tip of the snout to the vent that is seen in *E. antillensis*; *E. coqui* has tan/brown eyes (reddish in our individuals).

*Eleutherodactylus cochranae* Grant, 1932

Figure 3d

**Materials examined.** PUERTO RICO—**Arecibo** · Mata de Plátano Field Station and Nature Reserve; 18.414, −66.729; 151 m above sea level; Justin Matthew Bernstein, Camilo Andrés Calderón‐Acevedo, Pedro Ivo Mônico, Lázaro Willian Viñola‐Lopez, J. Angel Soto‐Centeno; Individual 1—found on east wall of field station at 9:10 PM.

**Identification.** One adult. Identified by its light coloration, two concave dorsal pigmentation lines, and a faint line of pigmentation in the middle of the snout.

**Lizards**

**Family Dactyloidae**

*Anolis cristatellus* Duméril and Bibron, 1837

Figure 4a

**FIGURE 4:**
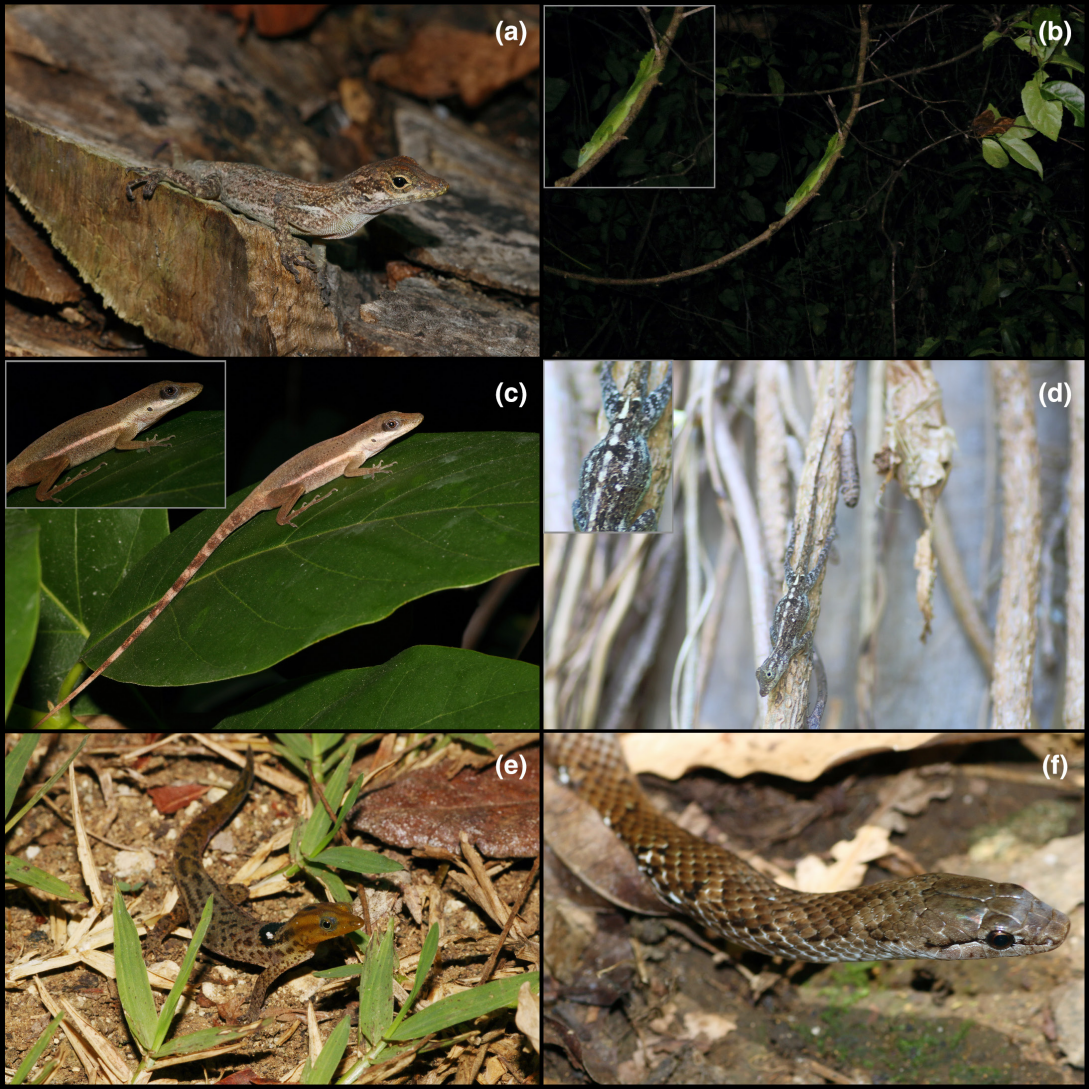
Lizards and snakes found at the Mata de Plátano Field Station and Nature Reserve. (a) *Anolis cristatellus*. (b) *Anolis cuvieri*, perched on a high branch; inset image zooms in on individual, showing the prominent crests and bright green color. (c) *Anolis kugri*; note the lateral lines and flecks of dark pigmentation on the flanks in the inset image. (d) *Anolis stratulus*; inset image better shows the saddle‐shaped blotches on the dorsum. (e) *Sphaerodactylus grandisquamis guarionex*. (f) *Borikenophis portoricensis*. All photographs taken by Justin M. Bernstein.

**Materials examined.** PUERTO RICO—**Arecibo** · Mata de Plátano Field Station and Nature Reserve; 18.414, −66.729; 151 m above sea level; Justin Matthew Bernstein, Camilo Andrés Calderón‐Acevedo, Pedro Ivo Mônico, Lázaro Willian Viñola‐Lopez, J. Angel Soto‐Centeno; Individual 1—found on karst wall on southwest side of field station at 6:00 PM; Individual 2–3—found on karst wall next to steel entrance gate of field station, 138 m southwest of field station building at 6:00 PM; Individuals 4–5—on walls, beneath second‐level deck of field station at 8:23 AM; Individual 6—Outside the entrance of Cueva Larvas (18.414° N, −66.727° W) in forest with cooler microclimate, found on vines with spotty sunlight, at 12:25 PM; Individuals 7–8—on palm tree at ~1.5 m off ground at 8:00 AM, performing territorial “push up” behavior; Individuals 9–10—on south wall of field station mating with each other at 4:58 PM; Individuals 11–13—sleeping on plants on forest edge on south side of field station at 12:30 AM; Individuals 14–17: found on plants on forest edge on south side of field station at 8:00 AM; Individuals 18–19 on branches ~1.5 m above ground, near karst wall on southwest side of field station at 2:45 PM; Individual 20—in forest on leaf litter, ~50 m south of Cueva de los Culebrones.

**Identification.** Identified based on morphology and geographic distribution. The brown/tan coloration, brown eyes, and crest of this species distinguish it from all other *Anolis* in Puerto Rico. *Anolis evermanni* has green coloration, and *A. stratulus* has large, dorsal, saddle‐shaped blotches on the back distinguished. *Anolis gundlachi* has blue eyes, *A. krugi* has yellow or cream‐colored lateral stripes (absent in our individuals), and *A. cuvieri* is a large, green crown giant anole. All other *Anolis* are found outside the range of Mata de Plátano.

*Anolis cuvieri* Merrem, 1820

Figure 4b

**Materials examined.** PUERTO RICO—**Arecibo** · Mata de Plátano Field Station and Nature Reserve; 18.414, −66.729; 151 m above sea level; Justin Matthew Bernstein, Camilo Andrés Calderón‐Acevedo, Pedro Ivo Mônico, Lázaro Willian Viñola‐Lopez, J. Angel Soto‐Centeno; Individual 1—found sleeping on U‐shaped vine ~4.5 m above ground at southeast edge of field station at 9:00 PM.

**Identification.** This species is readily distinguished from all congeners by being one of two crown giant anoles in the Puerto Rico Bank. Its bright green coloration, large dorsal body and tail crests, and large size (~125 mm; 5 mm snout‐vent‐length; Rivero, 1998) separate it from all other *Anolis*. The other crown giant, *A. roosevelti*, was distributed to the Puerto Rico satellite islands of Culebra and Vieques, and Tortola and St. John of the Virgin Islands, but is presumed to be extinct (Rivero, 1998).

*Anolis krugi* PETERS, 1877

Figure 4c

**Materials examined.** PUERTO RICO—**Arecibo** · Mata de Plátano Field Station and Nature Reserve; 18.414, −66.729; 151 m above sea level; Justin Matthew Bernstein, Camilo Andrés Calderón‐Acevedo, Pedro Ivo Mônico, Lázaro Willian Viñola‐Lopez, J. Angel Soto‐Centeno; Individual 1–2—found on plants on forest edge on south side of field station at 5:00 PM; Individual 3—on branch ~1.5 m above ground, over karst wall on southwest side of field station at 3:00 PM; Individual 4—on branch ~1.7 m above ground on forest edge ~50 m south of field station; Individual 5—in forest on ground, ~50 m south of Cueva de los Culebrones.

**Identification.** Distinguished from all other species by a combination of the presence of prominent yellow/cream‐colored lateral lines that extend from the eye to the groin (also found in *A. pulchellus*) and numerous black specks on the sides of the back and flanks (absent in *A. pulchellus*) and the absence of a brown band behind the eye. All other *Anolis* are found outside the range of Mata de Plátano or lack the lateral lines and black specks on the back and flanks.

*Anolis stratulus* Cope, 1861

Figure 4d

**Materials examined.** PUERTO RICO—**Arecibo** · Mata de Plátano Field Station and Nature Reserve; 18.414, −66.729; 151 m above sea level; Justin Matthew Bernstein, Camilo Andrés Calderón‐Acevedo, Pedro Ivo Mônico, Lázaro Willian Viñola‐Lopez, J. Angel Soto‐Centeno; Individual 1—On south wall of field station at 11:00 AM; Individuals 2–3—found on karst wall on southwest side of field station at 8:23 AM; Individuals 4–6—found on karst wall on southwest side of field station 11:07 AM; Individuals 7–8—sleeping on plants on forest edge on south side of field station at 12:30 AM.

**Identification.** Readily identified from all other *Anolis* on the island by the prominent saddle‐shaped blotched on the dorsum. The lack of a crest on the dorsum or tail distinguishes it from all other Puerto Rican anoles, with the exception of *A. evermanni* and *A. occultus*. *Anolis evermanni* has bright green coloration (tan/brown in our individuals) and *A. occultus* has a downward taping snout and a smaller body size and tail than *A. stratulus*; both *A. evermanni* and *A. occultus* lack saddle‐shaped blotches on the dorsum.

**Family Teiidae**

*Pholidoscelis exsul* (Cope, 1862)

**Materials examined.** PUERTO RICO—**Arecibo** · Mata de Plátano Field Station and Nature Reserve; 18.414, −66.729; 151 m above sea level; Justin Matthew Bernstein, Camilo Andrés Calderón‐Acevedo, Pedro Ivo Mônico, Lázaro Willian Viñola‐Lopez, J. Angel Soto‐Centeno; Individual 1–2—on ground of trail ~6 m south of field station at 8:40 AM; Individual 3—on ground of trail ~15 m south of field station.

**Identification.** *Pholidoscelis exsul* is one of few teiid lizards found in Puerto Rico. It is distinguished from its island conspecifics by a combination of morphology (color pattern), but is primarily identified by geographic distribution. *Pholidoscelis wetmorei* is only found in the southwest part of the island, *P. alboguttatus* is endemic to Mona Island, and *P. desechensis* endemic to Desecheo Island.

**Family Sphaerodactylidae**

*Sphaerodactylus grandisquamis guarionex* Stejneger, 1904

Figure 4e

**Materials examined.** PUERTO RICO—**Arecibo** · Mata de Plátano Field Station and Nature Reserve; 18.414, −66.729; 151 m above sea level; Justin Matthew Bernstein, Camilo Andrés Calderón‐Acevedo, Pedro Ivo Mônico, Lázaro Willian Viñola‐Lopez, J. Angel Soto‐Centeno; Individual 1—under palm trash on side of trail ~15 m south of field station at 10:30 PM; Individual 2—under palm trash on side of trail ~15 m south of field station at ~8:30 AM; Individual 3—found on top of leaf litter in open field 75 m south of field station at 6:20 PM; Individuals 4–10—found on top of leaf litter in open field 85 m south of field station at 6:30 PM; Individual 11—found on top of leaf litter in open field 85 m south of field station at 7:35 PM; Individuals 12–16—found on top of leaf litter in open field 85 m south of field station at 9:50 AM; Individuals 17–22—found on top of leaf litter in open field 85 m south of field station at 2:50 PM.

**Identification.** The only species of *Sphaerodactylus* in the northern part of mainland Puerto Rico are *S. g. guarionex* and *S. klauberi* (sensu Daza et al., 2019). *Sphaerodactylus g. guarionex* is distinguished from *S. klauberi* by its smaller size and lighter color (larger total length and snout‐vent‐length and dark, near‐black color in *S. klauberi*); *S. g. guarionex* is distinguished by other subspecies of *S. grandisquamis* by its reduced number of dorsal body scales (~14 for *S. g. guarionex*, 17–23 in *S. g. grandisquamis*, *S. g. spanius*, *S. g. mimetes*, and *S. g. ateles*) and rounded snout scales (Daza et al., 2019). Molecular and morphological data from recent studies support the claim that *Sphaerodactylus* in the northern part of the mainland are *S. g. guarionex* or *S. klauberi* (Daza et al., 2019; Reynolds et al., 2021).

**Snakes**

**Family Typhlopidae**

*Anillotyphlops* sp

**Materials examined.** PUERTO RICO—**Arecibo** · Mata de Plátano Field Station and Nature Reserve; 18.414, −66.729; 151 m above sea level; Justin Matthew Bernstein, Camilo Andrés Calderón‐Acevedo, Pedro Ivo Mônico, Lázaro Willian Viñola‐Lopez, J. Angel Soto‐Centeno; Individual 1—found in abandoned termite nest on side of trail ~2 m above the ground, ~125 m southwest of field station, at 11:00 PM. Individual quickly escaped and fell down an escarpment upon discovery.

**Identification.** Individual could not be identified to the species level, but is distinguished from all other snakes on the island by its smooth, shiny scales, extremely small size with cylindrical body (~15 cm total length), neck not visibly distinct from head, and the utilization of termite nest microhabitats.

**Family Dipsadidae**

*Borikenophis portoricensis* (Reinhardt and Lütken, 1862)

Figure 4f

**Materials examined.** PUERTO RICO—**Arecibo** · Mata de Plátano Field Station and Nature Reserve; 18.414, −66.729; 151 m above sea level; Justin Matthew Bernstein, Camilo Andrés Calderón‐Acevedo, Pedro Ivo Mônico, Lázaro Willian Viñola‐Lopez, J. Angel Soto‐Centeno; Individual 1—found under tarp from an old harp net bat trap on side of trail, ~10 m from the entrance of Cueva de los Culebrones, at 12:52 PM.

**Identification.** Easily diagnosed from blind snakes (*Anillotyphlops*, *Typhlops*), by its significantly larger size total length and circumference, and enlarged eyes. As a racer, it is not similar in appearance to *Xenocrophis* or *Chilabothrus*. *Borikenophis portoricensis* is distinguished from *Magliophis* by its larger size (*Magliophis* ~0.5 m; *Borikenophis* from this study ~1–1.2 m total length). *Borikenophis portoricensis* can be distinguished from its congeners by distribution and morphology: *B. prymnus* is smaller and paler in color and is found in southern Puerto Rico and *B. variegatus* is endemic to Mona Island.

**Family Boidae**

*Chilabothrus inornatus* (Reinhardt, 1843)

Figure 5a–g

**FIGURE 5:**
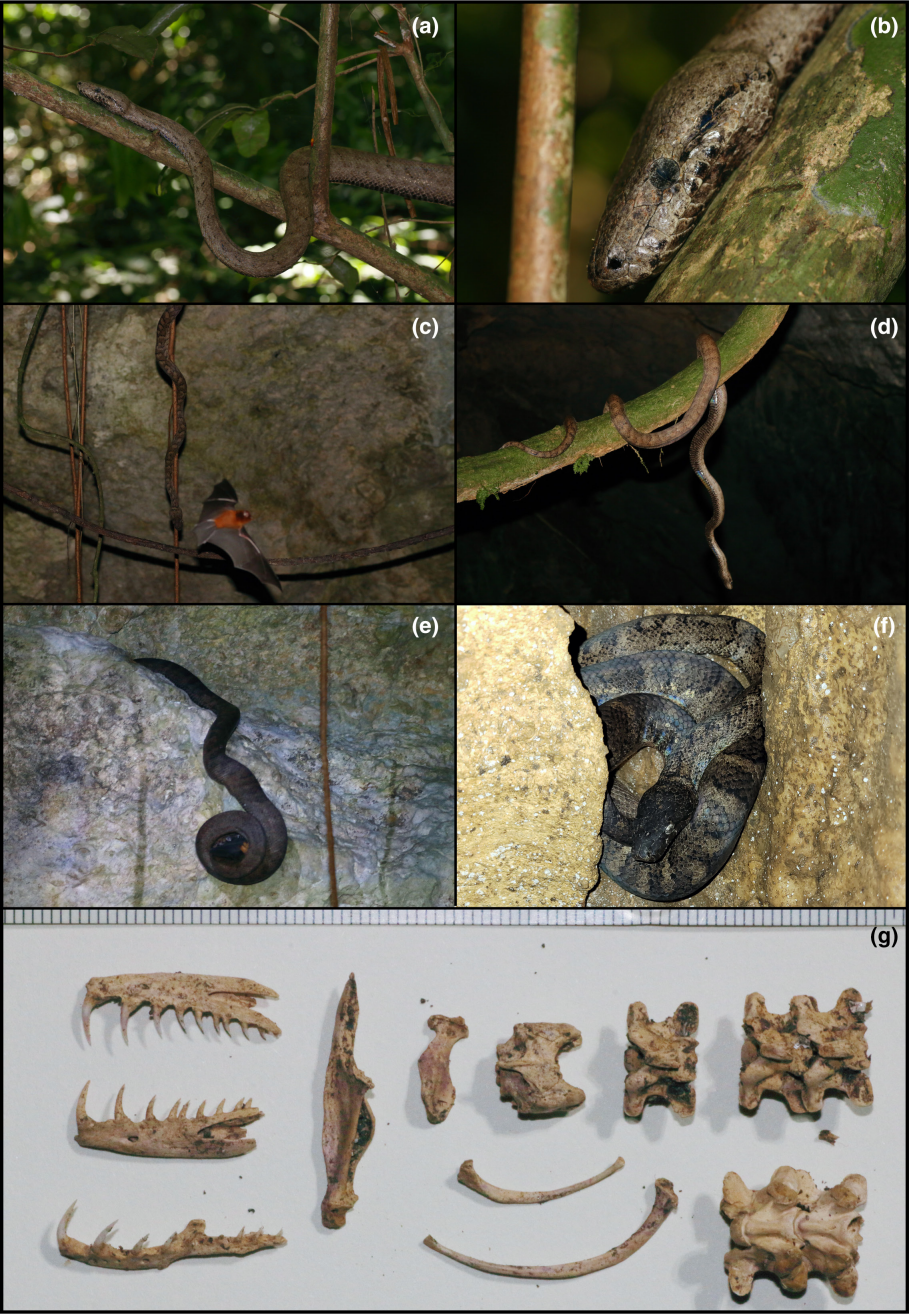
*Chilabothrus inornatus* from Cueva de los Culebrones. (a) Lateral view of *C. inornatus* perches on a branch. (b) Lateral shot of head, perched on a high branch. (c, d) Two different individuals of *C. inornatus* hanging vertically to catch bats flying out of the cave; Panel c shows an individual of *Mormoops blainvillei* flying in front of a Puerto Rican Boa. (e) A Puerto Rican Boa constricting its prey, *M. blainvillei*. (f) A dark pigmented individual of *C. inornatus* from the thermocline inside Cueva de los Culebrones. (g) Bones of *C. inornatus* found inside Cueva de los Culebrones. Cranial elements, vertebrae, and ribs were found. All photographs taken by Justin M. Bernstein.

**Materials examined.** PUERTO RICO—**Arecibo** · Mata de Plátano Field Station and Nature Reserve; 18.414, −66.729; 151 m above sea level; Justin Matthew Bernstein, Camilo Andrés Calderón‐Acevedo, Pedro Ivo Mônico, Lázaro Willian Viñola‐Lopez, J. Angel Soto‐Centeno; Individual 1–8—adult individuals found hanging on branches or on rock ledges of entrance of Cueva de los Culebrones, either perched or dangling vertically. One individual was observed eating *Mormoops blainvillei*. All individuals were seen at once at 7:00 PM; Individuals 9–11—juvenile individuals found hanging vertically from branches or rock ledges of entrance of Cueva de los Culebrones at 7:00 PM. All juveniles seen at the same time as Individuals 1–8; Individual 12—found inside Cueva de los Culebrones ~17.6 m depth moving along the ground; Individuals 13–14—adult skeletons, found at ~17.6 m depth in Cueva de los Culebrones; one skeleton ~30 m past cave entrance; second skeleton ~15 m before cave thermocline at ~20 m depth. Individuals 15–16—live adults, found coiled on limestone wall ledges ~1 m above the ground at the point of the thermocline inside Cueva de los Culebrones, at ~20 m depth.

**Identification.** As the only boa on Puerto Rico, this snake is unequivocally identifiable as *C. inornatus*. Its large, stout body, gray/silver scales, and the lack of labial thermic receptors are key characters of this species. *Chilabothrus inornatus* is distinguished from *C. granti* by the latter only being found on satellite islands (Culebra, and islets to the east) and *C. monensis* only found on Mona Island. Bones found in Cueva de los Culebrones were identified by the enlarged, recurved teeth characteristic of boids (Figure 5g); all other snakes on the island lack these and have much smaller teeth.

**FIGURE 6:**
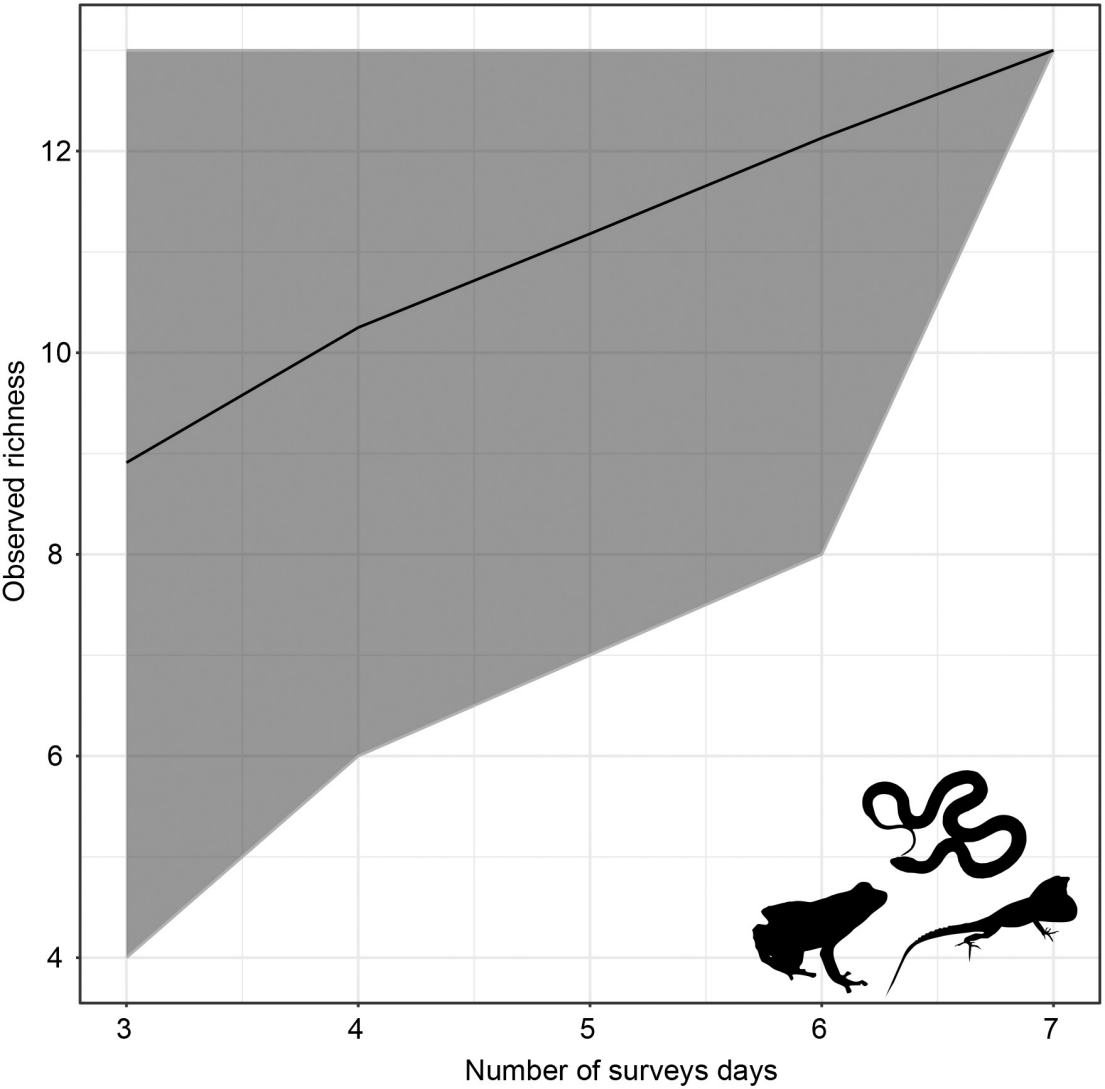
Species accumulation curve (SAC) for herpetofaunal species richness in this study. Black line represents observed species richness across number of survey days (gray shaded region = 95% confidence interval). Silhouettes obtained from PhyloPic.

For the herpetofauna, collectively, observed species richness increased with the number of surveys. The SAC calculated for the herpetofauna found in Puerto Rico strongly suggests that more sampling is needed, as evident from the lack of confidence intervals meeting the observed species richness curve (Figure 6).

**Chiroptera**

**Family Mormoopidae**

*Mormoops blainvillei* Leach, 1821

Figure 7a

**FIGURE 7:**
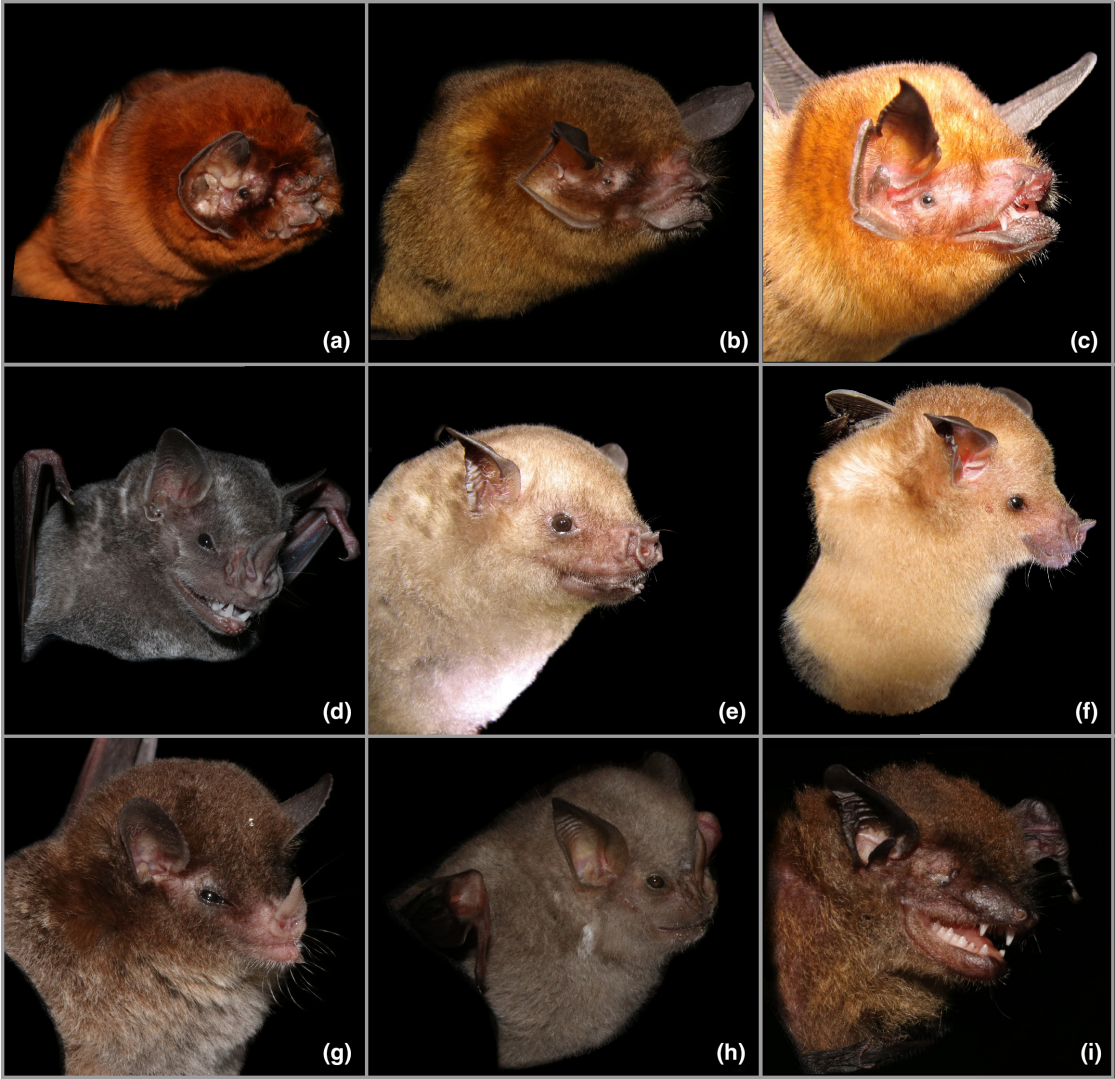
Bats caught in the mist nets at Mata de Plátano. Photos and parts of the body have been cropped out for easier viewing of headshots. (a) *Mormoops blainvillei*. (b) *Pteronotus portoricensis*. (c) *Pteronotus quadridens*. (d) *Artibeus jamaicensis*. (e) *Brachyphylla cavernarum*. (f) *Erophylla bombifrons*. (g) *Monophyllus redmani*. (h) *Stenoderma rufum*. (i) *Eptesicus dutertreus*. Photo credits: (a, b, d) Justin M. Bernstein; (c, e, f) J. Angel Soto‐Centeno.

**Materials Examined.** PUERTO RICO—**Arecibo** · Mata de Plátano Field Station and Nature Reserve; 18.414, −66.729; 151 m above sea level; Justin Matthew Bernstein, Camilo Andrés Calderón‐Acevedo, Pedro Ivo Mônico, Lázaro Willian Viñola‐Lopez, J. Angel Soto‐Centeno; captured in six‐meter mist net in open field 75 m south of field station between 6:45 PM and 8:30 PM; 1 ♂, ASC 1490; 1 ♀, ASC 1485.

**Identification.** The Antillean ghost‐faced bat (*M. blainvillei*) has a unique facial morphology that easily identifies it in the field. Nose‐leaf absent, snout is short and adorned with skin folds, the skin flap in the lower lip is split in two, giving the appearance of paired leaflets. Fur bright orange/reddish, the ears are joined by a membrane across the head. The ears are broad and round, with the outer rims connecting to the lower lip which gives the eyes the appearance of being immersed in the ear, inside a funnel. Size small, total length = 78–87 mm (tail length = 21 mm), (forearm = 45–48 mm) (Gannon et al., 2005; Wilson, 2023a).

*Pteronotus portoricensis* Miller, 1902

Figure 7b

**Materials Examined.** PUERTO RICO—**Arecibo** · Mata de Plátano Field Station and Nature Reserve; 18.414, −66.729; 151 m above sea level; Justin Matthew Bernstein, Camilo Andrés Calderón‐Acevedo, Pedro Ivo Mônico, Lázaro Willian Viñola‐Lopez, J. Angel Soto‐Centeno; captured in six‐meter mist net in open field 75 m south of field station between 6:45 PM and 8:30 PM; 1 ♂, ASC 1492.

**Identification.** The Puerto Rican mustached bat (*P. portoricensis*) has dark gray to grayish‐brown fur coloration, with ventral hairs being paler. Nose‐leaf absent, the snout protrudes more than in *M. blainvillei*, with the skin flap in the lower lip continuous and not split. The ears are pointed and with straight edges, not joined in the middle, eye position normal. It can only be confused with *P. quadridens*; however, it is considerably larger with a total length of 78–83 mm and a forearm over 44 mm. Additionally, it possesses a fleshy knob above the snout, which is lacking in *P. quadridens* (Gannon et al., 2005; Pavan, 2023).

*Pteronotus quadridens* Gundlach, 1840

Figure 7c

**Materials Examined.** PUERTO RICO—**Arecibo** · Mata de Plátano Field Station and Nature Reserve; 18.414, −66.729; 151 m above sea level; Justin Matthew Bernstein, Camilo Andrés Calderón‐Acevedo, Pedro Ivo Mônico, Lázaro Willian Viñola‐Lopez, J. Angel Soto‐Centeno; captured in six‐meter mist net in open field 75 m south of field station between 6:45 PM and 8:30 PM; 2 ♂, ASC 1484, ASC 1486; 3 ♀, ASC 1484, 1489, ASC 1491.

**Identification.** The sooty mustached bat (*P. quadridens*) has grayish‐brown fur with tricolored individual hairs. Nose‐leaf absent, as in *P*. portoricensis, the snout protrudes more than in *M. blainvillei*, with the skin flap in the lower lip continuous and not split. The ears are pointed and with straight edges, not joined in the middle, eye position normal. Considerably smaller than *P. portoricensis*, total length = 59–80 mm, forearm length = 36–39 mm. Can be discriminated from *P. portoricensis* by having a row of three to five small bulbs above each nostril, and the lack of a fleshy knob above the snout (Gannon et al., 2005; Pavan, 2023; Wilson, 2023b).

**Family Phyllostomidae**

*Artibeus jamaicensis* Leach, 1821

Figure 7d

**Materials Examined.** PUERTO RICO—**Arecibo** · Mata de Plátano Field Station and Nature Reserve; 18.414, −66.729; 151 m above sea level; Justin Matthew Bernstein, Camilo Andrés Calderón‐Acevedo, Pedro Ivo Mônico, Lázaro Willian Viñola‐Lopez, J. Angel Soto‐Centeno; captured in six‐meter mist net in open field 75 m south of field station between 6:45 PM and 8:30 PM; 1 ♂, ASC 1487; 1 ♀, ASC 1493.

**Identification.** Jamaican fruit‐eating bats (*A. jamaicensis*) were identified by their ashy‐gray coloration and faint but noticeable lighter color stripes on the rostrum. These bats have a broad nose leaf, a large central chin papillae surrounded by smaller ones, and a tail membrane that forms a V shape with no tail (Kwiecinski, 2023a). Jamaican fruit bats are medium‐sized, one of the stockier bats on the island, have broad dark brown wings, and are commonly captured on understory mist nets. We identified a small colony of Jamaican fruit bats present in Cueva Larvas.

*Brachyphylla cavernarum* Gray, 1834

Figure 7e

**Materials Examined.** PUERTO RICO—**Arecibo** · Mata de Plátano Field Station and Nature Reserve; 18.414, −66.729; 151 m above sea level; Justin Matthew Bernstein, Camilo Andrés Calderón‐Acevedo, Pedro Ivo Mônico, Lázaro Willian Viñola‐Lopez, J. Angel Soto‐Centeno; captured in six‐meter mist net in open field 75 m south of field station between 6:45 PM and 8:30 PM; 1 ♂, ASC 1487; 1 ♀, ASC 1493.

**Identification.** Antillean fruit‐eating bats (*B. cavernarum*) are medium‐sized and identified by their light brown to grayish pelage that is lighter in color at the base. These bats have a small and rudimentary nose‐leaf that almost give it a pig‐like appearance. Small papillae on the chin of Antillean fruit‐eating bats form a distinctive V shape (Kwiecinski, 2023b). The wings are broad and grayish in color. This species is a year‐round resident of Cueva de los Culebrones.

*Erophylla bombifrons* Miller, 1899

Figure 7f

**Materials Examined.** PUERTO RICO—**Arecibo** · Mata de Plátano Field Station and Nature Reserve; 18.414, −66.729; 151 m above sea level; Justin Matthew Bernstein, Camilo Andrés Calderón‐Acevedo, Pedro Ivo Mônico, Lázaro Willian Viñola‐Lopez, J. Angel Soto‐Centeno; observed roosting and flying in first open room of Cueva de los Culebrones.

**Identification.** Brown flower bats (*E. bombifrons*) have light brown to yellowish colored fur that is very short. We identified roosting and flying brown flower bats at Cueva de los Culebrones by their characteristic hair color, which easily reflects light. On the one hand, brown flower bats are small bats with a slightly elongated and semi‐naked rostrum. (Speer, 2023) Their nose leaf is small and pointy. They have a V‐shaped tail membrane and a tail shorter than the femur extends beyond the membrane. These bats are abundant at Cueva de los Culebrones and often are hunted by the Puerto Rican boa. Although no individuals were captured in mist nets, brown flower bats can be captured in understory mist nets set close to fruiting Panama berry trees (*Mutingia calabura*), piper plants (*Piper aduncum*), or turkey berry plants (*Solanum torvum*). This species is a year‐round resident of Cueva de los Culebrones.

*Monophyllus redmani* Leach, 1821

Figure 7g

**Materials Examined.** PUERTO RICO—**Arecibo** · Mata de Plátano Field Station and Nature Reserve; 18.414, −66.729; 151 m above sea level; Justin Matthew Bernstein, Camilo Andrés Calderón‐Acevedo, Pedro Ivo Mônico, Lázaro Willian Viñola‐Lopez, J. Angel Soto‐Centeno; captured in six‐meter mist net in open field 75 m south of field station between 6:45 PM and 8:30 PM; 1 ♂, ASC 1488.

**Identification.** The Greater Antillean long‐tongued bat (*M. redmani*) is easily identified by being the smallest leaf‐nosed bat on the island. This primarily nectarivorous species has grayish brown pelage, sometimes with patches of white hairs present on the dorsal side. The ventral side is lighter grayish brown in color. This bat has an elongated rostrum culminating in a distinctive arrow‐shaped nose leaf (Kurta & Rodríguez‐Durán, 2023). The tail membrane is V‐shaped and a short tail extends beyond the tip of the membrane. Greater Antillean long‐tongued bats are captured in understory mist nets near fruiting Panama berry trees (*M. calabura*) and flowering endemic Maga trees (*Thespesia grandiflora*). This species is a year‐round resident of Cueva de los Culebrones.

*Stenoderma rufum* Desmarest, 1820

Figure 7h

**Materials Examined.** PUERTO RICO—**Arecibo** · Mata de Plátano Field Station and Nature Reserve; 18.414, −66.729; 151 m above sea level; Justin Matthew Bernstein, Camilo Andrés Calderón‐Acevedo, Pedro Ivo Mônico, Lázaro Willian Viñola‐Lopez, J. Angel Soto‐Centeno; captured in six‐meter mist net in open field 75 m south of field station between 6:45 PM and 8:30 PM; 1 ♂, ASC 1494.

**Identification.** The red fig‐eating bat (*S. rufum*) is the only other member of the subfamily Stenodermatinae inhabiting Puerto Rico. Nose leaf present and broad, as in *A. jamaicensis*, its fur is tan to dark chocolate‐brown (its conspecifics inhabiting St. Croix and St. John having a reddish hue in their fur), the venter is lighter than the back, and it is the only bat in Puerto Rico with distinctive white epaulets. Although it is similar to *A. jamaicensis*, it can be diagnosed by its smaller size, total length = 60–73 mm, and forearm length = 46–52 mm. Additionally, it lacks any facial stripes (Gannon et al., 2005; Genoways, 2023). The connectivity and abundance of its populations are affected by seasonal climatic disturbances like hurricanes (Calderón‐Acevedo et al., 2021; Gannon & Willig, 1994).

**Family Vespertilionidae**

*Eptesicus dutertreus* Gervais, 1837 (Mônico & Soto‐Centeno, 2024; *Eptesicus fuscus*, op cit.)

Figure 7i

**Materials Examined.** PUERTO RICO—**Arecibo** · Mata de Plátano Field Station and Nature Reserve; 18.414, −66.729; 151 m above sea level; Justin Matthew Bernstein, Camilo Andrés Calderón‐Acevedo, Pedro Ivo Mônico, Lázaro Willian Viñola‐Lopez, J. Angel Soto‐Centeno; captured in six‐meter mist net in open field 75 m south of field station between 6:45 PM and 8:30 PM; 1 ♂, ASC 1483.

**Identification.** The identity of Greater Antillean serotine bats (*E. dutertreus*) was confirmed by their characteristically dark chocolate brown fur, and fully naked nearly black round ears, wing, and tail membranes. Both the tail and wing membranes look oily. The rostrum of Greater Antillean serotines is naked and wide, giving it an appearance of being inflated, a trait not found in other bats in Puerto Rico. These bats are fast fliers that forage above the canopy of trees. Our capture of Greater Antillean serotine bats in a forest corridor using understory mist nets is attributed to the presence of Cueva Larvas that serves as a roost nearby. We note that we use the new name combination *Eptesucus dutertreus*, which applies to all big brown bats from the Bahamas and the Greater Antilles (see Mônico & Soto‐Centeno, 2024).

## 4 DISCUSSION

Mata de Plátano Field Station and Nature Reserve (MPFS) is best known for its bat‐snake interactions, yet no faunal surveys for either of these groups have been published. Our study shows that while small (~5100 m^2^), this reserve has a rich herpetofauna and bat fauna representative of the overall diversity of the island. This rapid inventory provides the first local, taxonomic‐focused checklist for these species in north‐central Puerto Rico.

**FIGURE 8:**
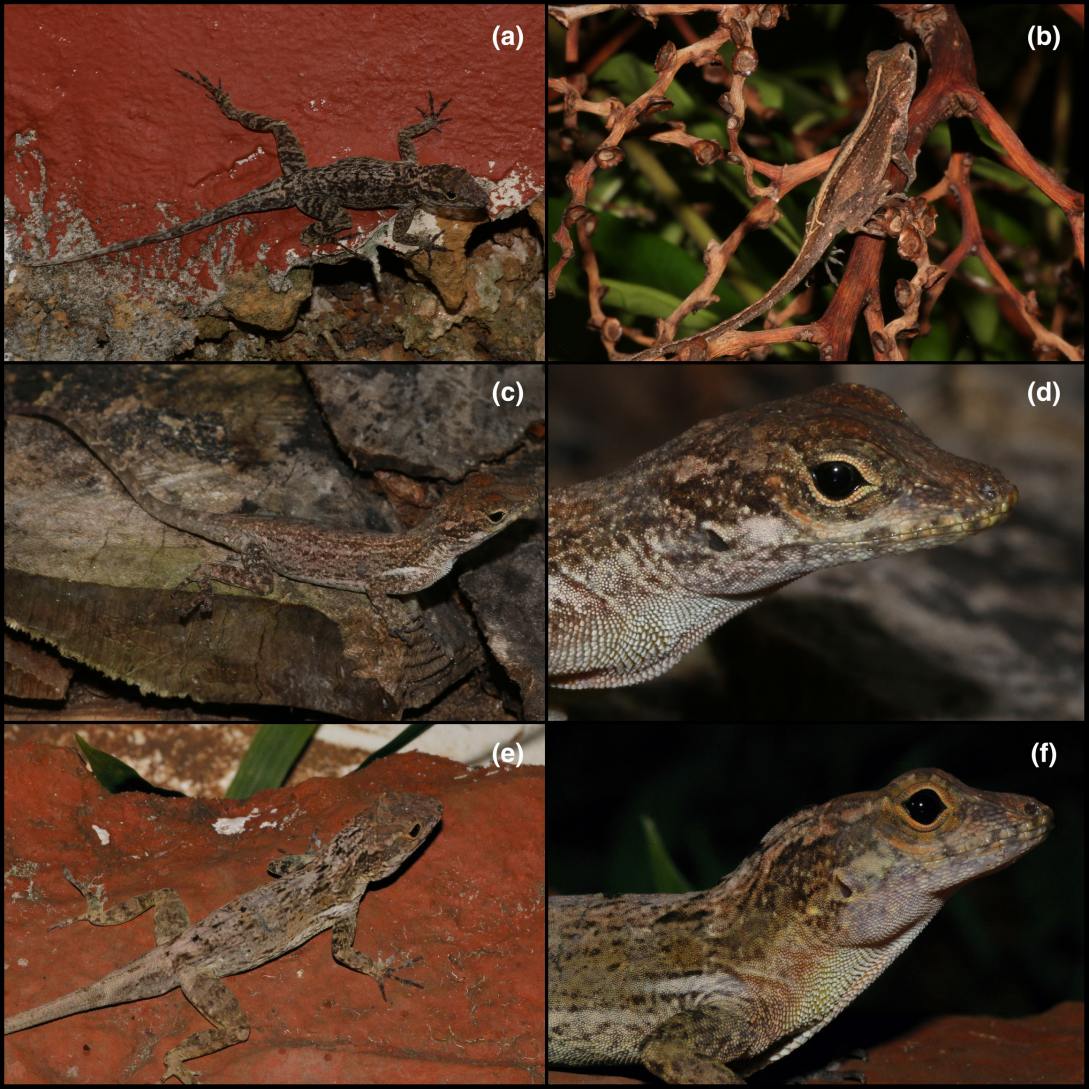
Variation seen in *Anolis cristatellus* from the Mata de Plátano Field Station. (a) Gray/tan patterning with heavy mottling. (b) Brown morph with mid‐dorsal yellow stripe and light dorsal and lateral flecks. (c) Brown morph with no mottling or mid‐dorsal spots, light flecks present. (d) Lateral shot of the head of the individual from Panel C. (e) Brown morph with light mottling and striping, and dark spots running down the spine. Note the slightly raised dorsal crest (more prominent at the tail). (f) Lateral shot of the head of the individual from Panel f. All photographs taken by Justin M. Bernstein.

In our 10‐day rapid inventory, we found a total of 14 species of reptiles and amphibians. We documented four amphibians, seven lizards, and three snakes, totaling up to ~22% (four out of 18) and ~ 14% (10 out of 72) of the amphibians and squamate diversity on Puerto Rico. Our observations during field work also provide important natural history information. We documented *A. cristatellus* copulating on the field station walls, which is not surprising as this species is often found in man‐made structures throughout the island and is abundant in urban areas. Additionally, we documented two morphs of *A. cristatellus*: (1) light gray/tan (with or without darker mottling), and (2) a morph that is dark or light brown with a light yellow or cream‐colored mid‐dorsal stripe and light‐colored dorsal and lateral flecks. The dorsum of the first morph sometimes has dark gray or black spots running down the spine but are not nearly as distinct or as large as the saddle‐shaped botched of *A. stratulus* (Figure 8). The dorsal crest and tail fin in both morphs are sometimes prominent, while other observations the cutest was hardly distinct from the rest of the body. We also report notes on the endemic *Chilabothrus inornatus*. This species is known for consumption of bats on the island, and many individuals aggregate at the entrance and inside caves to opportunistically catch them. We saw one adult individual attack and devour a ghost‐faced bat (*Mormoops blainvillei*). In our night surveys, we observed juvenile *C. inornatus* snakes (*N* = 3) at the entrance of Cueva de los Culebrones, which suggests an actively breeding population of this endemic snake. We also found three live (and an additional two separate skeletons) *C. inornatus* inside the cave, at 17.6 and ~20 m depth. The latter of these two depths is the thermocline of the cave (~35°C), where we found two live *C. inornatus*. Individuals found deeper in the cave range from light gray to dark, charcoal gray (Figure 5f). Compared to the ambient temperature (average: 24.4–31.1 °F), the endemic Puerto Rican Boas tolerate a wide range of temperatures.

During this rapid inventory, we observed nine species of bat, all of which were documented in a single 1.75‐h period of mist netting. This bat diversity represents 70% (i.e., nine of 13) of the living alpha diversity present on the island of Puerto Rico. Most of the bats we observed are residents of MPFS. While our sampling efforts do not permit a more thorough examination of species abundance or comparisons with other reserves in the island, these data highlight the importance of MPFS as a local center of bat diversity. The moist forest area where MPFS is located is adjacent to pastures, unprotected rustic forest areas, and the urbanized center of the municipality of Arecibo to the north. However, more importantly, the station connects with the larger Río Abajo State Forest to the south. Together these two protected areas form a hub in the northcentral part of Puerto Rico extending from coastal to mountainous areas and linking multiple forested corridors that connect with reserves along the Karst Conservation Zone (Calderón‐Acevedo et al., 2021).

Two endemic bat species documented in this rapid survey are of particular importance. The endemic Puerto Rican mustached bat (*Pteronotus portoricensis*) specifically roosts in hot cave chambers where temperatures often exceed 35°C, such as those present in Cueva de los Culebrones (Rodríguez‐Durán & Soto‐Centeno, 2003). Previous studies show that the population of this species at Cueva de los Culebrones was not affected by hurricanes (Jones et al., 2001). Nonetheless, *P. portoricensis* seems to be a new resident to the cave, and thus effects of natural phenomena on this species at this locality are not well known. A single individual of another endemic, the red fig‐eating bat (*Stenoderma rufum*), was captured during our survey. This endemic frugivorous bat is known to roost in the thick foliage of trees and occurs in low abundance throughout the island (Calderón‐Acevedo et al., 2021; Genoways, 2023). Given these characteristics, *S. rufum* is a species particularly affected by strong hurricanes (Gannon & Willig, 1994), which can decrease the structural habitat connectivity through the island. The protection of MPFS in the wider northcentral habitat connectivity hub of Puerto Rico (Calderón‐Acevedo et al., 2021), may be key for the recovery and population connectivity of this rare bat.

To our knowledge, this is the first faunal checklist on the bat and herpetofauna of Puerto Rico. Our findings expand the current knowledge of biodiversity and species richness in this protected region. We acknowledge that while our species accumulation curve suggests that more surveys are needed to determine species richness in this area, our sampling period is short and varied by day. More standardized methods over greater periods of time will provide more insight on species abundances. Nonetheless, we provide valuable biodiversity information here. Our results show that *A. cristatellus* and *S. grandisquamis guarionex* likely contribute large portions to the vertebrate biomass here, as has been found in other studies (Rodda et al., 2001). Future sampling should also target specific microhabitats for reported species. For example, there have been several accounts of *A. occultus* from the field station (pers. comm. Abel Vale). Although we did not see these species during our time at the reserve, future surveys will be needed to better understand the density of *A. occultus* and *N. leporinus* in this area. The Puerto Rican fauna has been studied well, yet new species have recently been described (e.g., Díaz‐Lameiro et al., 2022), thus it is critical to perform local, intensive surveys and document faunal inventories. These local intensive surveys and faunal checklists can provide a steady supply of data to understand the current status of Caribbean species. The main threats to Caribbean bats and herpetofauna stem from habitat loss and fragmentation related to human development, and climatic events that can drastically re‐shape the local population connectivity of bats species (Calderón‐Acevedo et al., 2021; Soto‐Centeno & Calderón‐Acevedo, 2023). Therefore, having updated local lists can provide a year‐to‐year comparison of local faunal abundance that can inform conservation strategies (Joglar et al., 2011), and eventually strengthen the predictive power when modeling the effect climate change of species richness and abundance. We encourage more researchers to publish faunal and floral lists, even for common taxa, to better understand species diversity, abundance, and interactions for downstream research and conservation.

## AUTHOR CONTRIBUTIONS

**Justin Matthew Bernstein:** Conceptualization (lead); data curation (equal); formal analysis (equal); investigation (equal); methodology (equal); resources (supporting); visualization (lead); writing – original draft (lead); writing – review and editing (equal). **Camilo Andrés Calderón‐Acevedo:** Investigation (equal); methodology (equal); writing – review and editing (equal). **Pedro Ivo Mônico:** Investigation (equal); methodology (equal); writing – review and editing (supporting). **Lázaro Willian Viñola‐Lopez:** Investigation (equal); methodology (equal); writing – review and editing (supporting). **J. Angel Soto‐Centeno:** Data curation (equal); funding acquisition (lead); investigation (equal); methodology (equal); resources (equal); supervision (equal); visualization (supporting); writing – original draft (supporting); writing – review and editing (equal).

## CONFLICT OF INTEREST STATEMENT

The authors of this manuscript have no conflicts of interest.

## Data Availability

All researches here were observational, and no data beyond what is presented in this manuscript are available.
